# Predicting recurrence risk in endometrial cancer: a multisequence MRI intratumoral and peritumoral radiomics nomogram approach

**DOI:** 10.3389/fonc.2025.1569729

**Published:** 2025-05-06

**Authors:** Jie Li, Dianpei Ma, Xiuting Chen, Junting Wei, Jiali Xu, Yingming Zhao, Zhizhen Gao

**Affiliations:** ^1^ School of Medical Imaging, Bengbu Medical University, Bengbu, Anhui, China; ^2^ Department of Radiology, The First Affiliated Hospital of Bengbu Medical University, Bengbu, Anhui, China; ^3^ The First Affiliated Hospital of University of Science and Technology of China (USTC), Division of Life Sciences and Medicine, University of Science and Technology of China, Hefei, Anhui, China

**Keywords:** magnetic resonance imaging, peritumoral radiomics, machine learning, endometrial cancer, recurrence

## Abstract

**Objective:**

To assess the predictive value of a nomogram model incorporating clinical factors and multisequence MRI intratumoral and peritumoral radiomics features for estimating recurrence risk in endometrial cancer (EC) patients.

**Materials and methods:**

This retrospective study included 184 patients with EC. The samples were randomly divided into a training set and a test set according to a 7:3 ratio, and intratumoral and peritumoral radiomics features were extracted from diffusion-weighted imaging (DWI) and T2-weighted imaging (T2WI) sequences. Optimal radiomics features were selected using the f-classification function, minimum redundancy maximum relevance (mRMR) method, and least absolute shrinkage and selection operator (Lasso). Nine machine learning classifiers were employed to construct the intratumoral model (RM1). The best-performing classifiers were then used to develop the intratumoral and peritumoral 2 mm radiomics model (RM2) and the intratumoral and peritumoral 4 mm radiomics model (RM3). The radiomics scores (Rad-score) from the top-performing radiomics model were combined with clinical factors to create the nomogram model (FM). The predictive performance of the FM model was evaluated using receiver operating characteristic (ROC) curve analysis, calibration curve assessment, clinical decision curve analysis (DCA), clinical impact curve (CIC), and the DeLong test. Feature importance analysis using the SHapley Additive exPlanations (SHAP) methodology.

**Results:**

The logistic regression classifier (LR) showed optimal predictive efficacy, and RM2 demonstrated the best diagnostic performance. The clinical decision curve and DeLong test results indicated that the FM model was the optimal recurrence model in EC patients.

**Conclusion:**

A nomogram model integrating MRI radiomics features from intratumoral and peritumoral regions and clinical factors effectively predicts recurrence in EC patients.

## 1 Introduction

Endometrial cancer (EC) is the sixth most common cancer among women, with 417,000 new cases diagnosed globally in 2020. Its incidence and disease-related mortality rates have been on the rise globally, posing a significant threat to women’s health (1, 2). Research indicates that 10 to 20 percent of patients will experience a relapse within three years, and more than 95 percent will have a recurrence within five years (3). While progress has been made in comprehensive care for recurrent EC, overall effectiveness remains unsatisfactory, and the prognosis is poor (4). Therefore, it is increasingly crucial to predict recurrence in patients with EC shortly after surgery and to develop new biomarkers that can facilitate personalized treatment strategies.

Magnetic resonance imaging (MRI) offers unique advantages for soft tissue imaging and has been an effective tool for differential diagnosis and monitoring recurrence for an extended period. Radiomics is a rapidly developing discipline that offers extensive information about the phenotype and microenvironment of tumors through the quantitative analysis of medical imaging features such as intensity, shape, size, volume, and texture. These quantitative image features complement routine clinical reports, laboratory test results, and genomic or proteomic analyses, providing new insights for clinical decision-making (5–7). Numerous studies have employed radiomics to investigate various aspects of EC. However, there has been less emphasis on studying the recurrence of EC (8). A nomogram, as a graphical tool for predicting individualized clinical event probabilities by integrating the effects of multiple variables, fulfills the requirement for comprehensive clinical models and provides robust support for personalized medicine (9). While the majority of studies have concentrated on the tumor itself, some have incorporated information about the environment surrounding the tumor to predict vascular invasion and lymph node metastasis, yielding superior results (10). Furthermore, certain studies have already included the peritumoral area as a significant component in the extraction of radiomics features to predict recurrence in patients with EC (11).

The objective of this study was to combine clinical factors with radiomics features obtained from MRI to create a nomogram model. This model is designed to predict the risk of recurrence in EC patients, ultimately guiding precision medicine and personalized treatment strategies.

## 2 Materials and methods

### 2.1 Study population

This study received approval from the Medical Ethics Committee of the First Affiliated Hospital of Bengbu Medical University. Furthermore, the study was exempt from the requirement for informed consent from the subjects. Data from patients who met the following criteria between January 2019 and December 2021 were retrospectively collected and analyzed for this study. Based on the inclusion and exclusion criteria, this retrospective study included 184 patients with EC. The inclusion criteria were as follows: (1) EC was confirmed based on clinical presentation, auxiliary examinations, and postoperative histopathological examination; (2) pelvic-enhanced MRI was performed within two weeks prior to surgery; and (3) there was no history of other malignant tumors. The exclusion criteria included: (1) follow-up of less than three years; (2) incomplete clinical or imaging data; (3) previous biopsy, surgery, or radiotherapy before the examination; (4) lack of usable lesion tissue to outline the region of interest (ROI); (5) poor image quality or presence of artifacts; and (6) International Federation of Gynecology and Obstetrics (FIGO) stage IV. Ultimately, data from 184 patients with EC cases were included in the analysis. Patients were followed up for a minimum of 36 months. The specific follow-up procedures and definitions of recurrence are provided in Appendix E1.

### 2.2 Clinical information incorporated

A comprehensive dataset, including clinical, pathological, and laboratory information, was retrieved from the electronic medical record system. The following variables were considered: age, age at menarche, menopausal status, reproductive history, hypertension, diabetes mellitus, International Federation of Gynecology and Obstetrics (FIGO) stage, pathological type, pathological grade, myometrial invasion, lymph node metastasis, radiation therapy, chemotherapy, surgical procedure, and laboratory values such as absolute pre-operative neutrophil count (N) (×10^9^/L), absolute preoperative lymphocyte count (L) (×10^9^/L), preoperative fibrinogen (FIB) levels, preoperative serum cancer antigen 125 (CA125) levels, and postoperative serum CA125 levels. The preoperative indices were obtained from examinations conducted one week before surgery, while the postoperative indices were gathered from follow-up examinations conducted 6 to 12 months after surgery. The pathological type was categorized into two classifications: endometrioid endometrial carcinoma (EEC) and non-endometrioid endometrial carcinoma (NEEC), according to the commonly accepted dichotomy (12). The pathological grading of the tumor was determined by assigning grades 1 and 2 as low grade and grade 3 as high grade. The neutrophil-lymphocyte ratio (NLR) was calculated using the following formula: *NLR* = *N/L*.

### 2.3 Tumor segmentation

The image Acquisition parameters are provided in Appendix E2. The images obtained were imported into the DARWIN Intelligent Research Platform (http://www.yizhun-ai.com). The DARWIN Intelligent Research Platform is an end-to-end integrated tool dedicated to AI-powered medical imaging research, designed to lower the barriers for clinicians engaging in AI medical studies by enabling efficient completion of full-cycle research workflows from data management to research output generation. Two radiologists with over five years of experience in gynecological malignancies independently delineated the three-dimensional volumes of interest (VOIs) in the sequence at one-week intervals, while being blinded to the patient’s pathological information. The T2WI sequence was subsequently matched with the DWI sequence, and VOIs were delineated based on the orientations of the lesions in the different sequences. All annotations were carefully documented (**Supplementary Figure S1**). The peritumoral VOIs were obtained by the platform automatically expanding 2mm and 4mm. When discrepancies existed, consensus was reached through joint discussion or determined by a senior physician.

### 2.4 Feature filtering and dimensionality reduction

After contouring all patient VOIs, we extracted and analyzed the radiomics features. Intraclass correlation coefficient (ICC) was calculated to assess both the intra-observer and inter-observer reproducibility of radiomics features, with features retaining ICC values ≥0.8. To eliminate the dimensional differences among these features, we applied the max-min normalization method to transform each feature to a (0, 1) range. Next, we selected the optimal feature screening method (percentage) to reduce the number of features. The f-classif function was used to assess the linear correlation between features and recurrence. Specifically, the top 50% of features were retained for the intratumoral analysis, while the top 25% of features were retained for both the intratumoral and peritumoral 2 mm and the intratumoral and peritumoral 4 mm analyses. For further feature selection, we used the minimum redundancy maximum relevance (mRMR) method, which aims to maximize the correlation between features and recurrence while minimizing redundancy among them. We selected the top 50 variables most correlated with recurrence based on the mutual information difference method and removed features that exhibited high inter-feature correlation. Finally, the least absolute shrinkage and selection operator (LASSO) regression was utilized to identify the most valuable features for predicting recurrence in EC patients.

### 2.5 Machine learning model development

After the completion of the feature screening, we imported the optimal intratumoral radiomics features into nine different classifiers to develop an intratumoral radiomics model (RM1). The following classifiers were considered: support vector machine (SVM), random forest (RF), extreme gradient boosting (XGBoost), logistic regression (LR), decision tree (DT), light gradient boosting machine (lightGBM), adaptive boosting (AdaBoost), gradient boosting decision tree (GBDT), and K nearest neighbors (KNN). The objective of this step is to compare the classification performance of multiple classifiers and identify the best one for constructing both the intratumoral and peritumoral 2mm radiomics model (RM2) and the intratumoral and peritumoral 4mm radiomics model (RM3). The reliability of these models was verified using ten-fold nested cross-validation, and the radiomics score (Rad-score) was obtained, respectively. Following the completion of model construction, the area under the curve (AUC) of the receiver operating characteristic (ROC) curve was compared among the three models to identify which radiomics model demonstrated the best predictive performance. The clinical information of the patients was initially analyzed using univariate analysis. The variables that proved significant in this analysis were then included in a multivariate analysis. Factors with a statistically significant p-value (p < 0.05) were retained for constructing the clinical prediction model (CM). Ten-fold nested cross-validation was also used to assess the stability of the model. The final step involved creating a nomogram that integrated clinical independent risk factors alongside the Rad-score, which was then visually rendered.

### 2.6 Statistical analysis

The missing data rates were 4.348% for pathological grade, 6.522% for the NLR, 6.522% for serum CA125, and 3.261% for postoperative serum CA125. Continuous variables with missing values were imputed using the mean, while categorical variables with missing data were imputed using the mode. Statistical analysis was carried out using SPSS software (version 27.0). The non-statistical analysis was conducted using the R software (version 4.3.2). Wilcoxon rank sum test, Pearson’s Chi-squared test, and Fisher’s exact test were used to analyze the differences between the training group and the validation group. A two-tailed p < 0.05 was considered statistically significant.

## 3 Results

### 3.1 Comparison of clinical information

**Figure 1:**
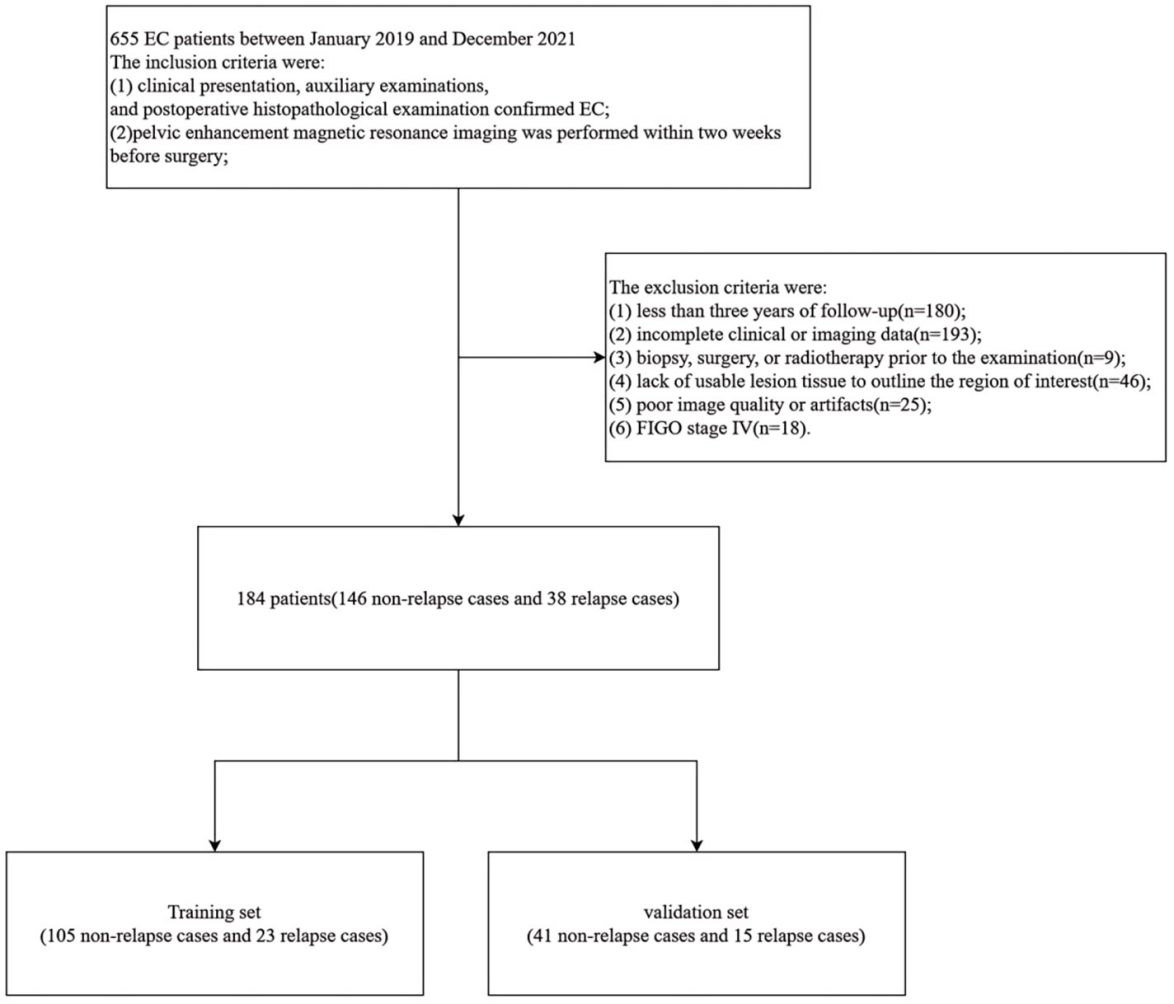
Patient screening flowchart.

**Figure 2:**
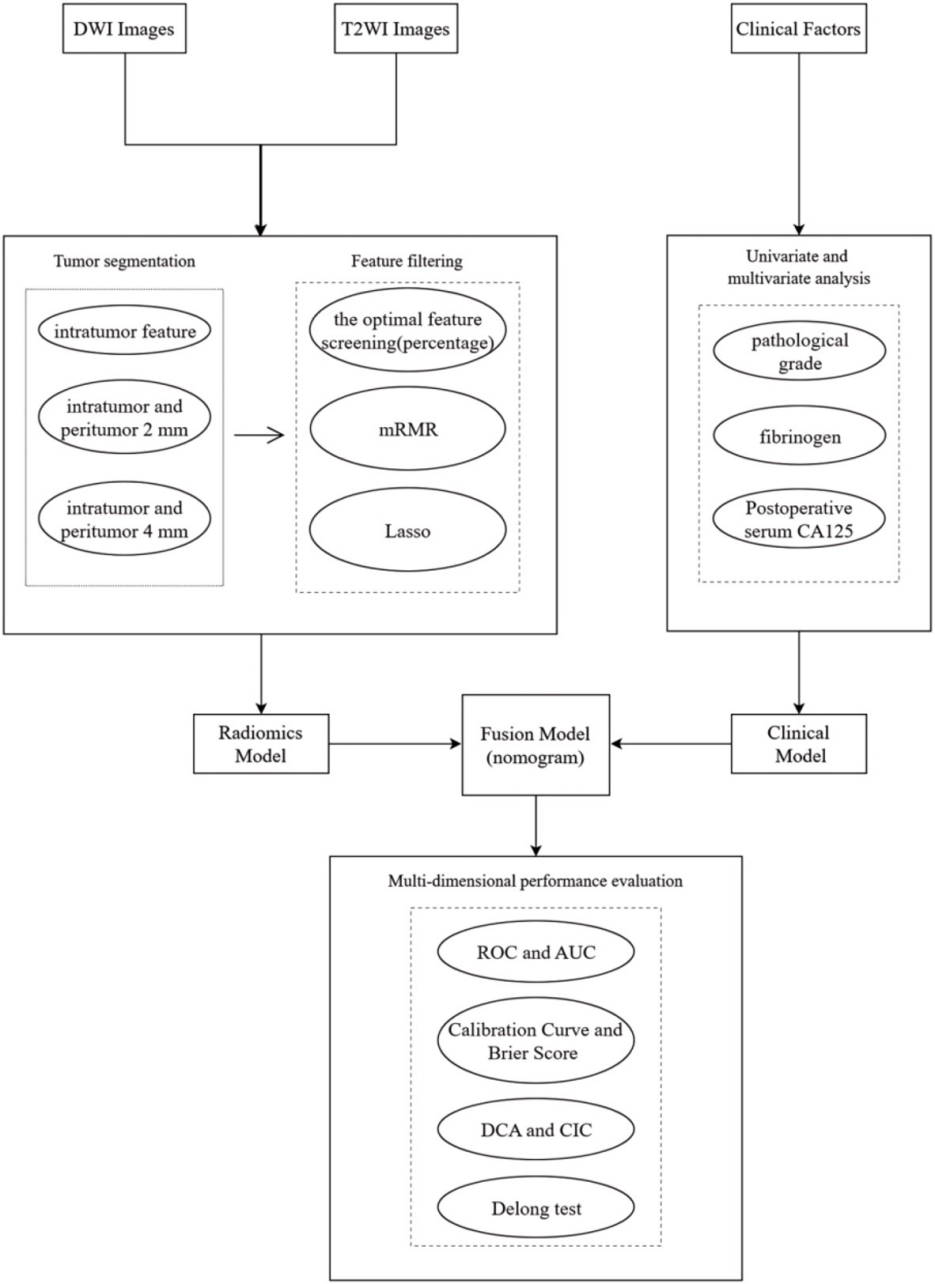
Experiment flowchart.

A total of 184 patients with EC were enrolled in this study (**Figure 1**), consisting of 146 non-relapse cases and 38 relapse cases. The patients were randomly divided into a training set and a validation set in a 7:3 ratio. The training set included 105 non-relapse cases and 23 relapse cases, while the validation set contained 41 non-relapse cases and 15 relapse cases. Statistical analysis indicated that the distribution of relevant clinical factors in both the training and validation sets did not differ significantly (p > 0.05) (**Table 1**). **Figure 2** depicts the flow of the experiment.

**Table 1:** Comparison of clinical information in the training and validation sets of endometrial cancer patients.

Variable	Group			p-value^2^
	Overall N = 184^1^	Training set N = 128^1^	Validation set N = 56^1^	
**Age**	54.00 (51.00–58.00)	53.50 (50.00–57.00)	56.00 (51.500–62.00)	0.036
**Age at menarche**	12.00 (12.00–13·00)	12.00 (12.00–14.00)	12.00 (12.00–12.00)	0.041
**Menopausal status**				0.764
No	72 (39%)	51 (40%)	21 (38%)	
Yes	112 (61%)	77 (60%)	35 (63%)	
**Reproductive history**				0.517
No	2 (1.1%)	1 (0.8%)	1 (1.8%)	
Yes	182 (99%)	127 (99%)	55 (98%)	
**Hypertensive**				0.444
No	116 (63%)	83 (65%)	33 (59%)	
Yes	68 (37%)	45 (35%)	23 (41%)	
**Diabetes**				0.079
No	160 (87%)	115 (90%)	45 (80%)	
Yes	24 (13%)	13 (10%)	11 (20%)	
**FIGO stage**				0.379
I	142 (77%)	95 (74%)	47 (84%)	
II	33 (18%)	26 (20%)	7 (13%)	
III	9 (4.9%)	7 (5.5%)	2 (3.6%)	
**Pathological type**				0.249
EEC^3^	176 (96%)	124 (97%)	52 (93%)	
NEEC^4^	8 (4.3%)	4 (3.1%)	4 (7.1%)	
**Pathological grade**				0.617
Low	158 (86%)	111 (87%)	47 (84%)	
High	26 (14%)	17 (13%)	9 (16%)	
**Myometrial invasion**				0.168
None	9 (4.9%)	8 (6.3%)	1 (1.8%)	
<1/2	136 (74%)	97 (76%)	39 (70%)	
≥1/2	39 (21%)	23 (18%)	16 (29%)	
**Lymph node metastasis**				0.844
No	163 (89%)	113 (88%)	50 (89%)	
Yes	21 (11%)	15 (12%)	6 (11%)	
**Radiation therapy**				0.690
No	151 (82%)	106 (83%)	45 (80%)	
Yes	33 (18%)	22 (17%)	11 (20%)	
**Chemotherapy**				0.614
No	54 (29%)	39 (30%)	15 (27%)	
Yes	130 (71%)	89 (70%)	41 (73%)	
**Recurrence**				0.174
No	146 (79%)	105 (82%)	41 (73%)	
Yes	38 (21%)	23 (18%)	15 (27%)	
**Surgical procedure**				0.219
Laparoscopic surgery	98 (53%)	72 (56%)	26 (46%)	
Laparotomy	86 (47%)	56 (44%)	30 (54%)	
**NLR** ^5^				0.208
≤1.765	109 (59%)	79 (62%)	30 (54%)	
>1.765	75 (41%)	49 (38%)	27 (46%)	
**Fibrinogen**				0.393
≤3.790	151 (82%)	103 (80%)	48 (86%)	
>3.790	33 (18%)	25 (20%)	8 (14%)	
**Preoperative serum CA125**				>0.999
≤233.700	177 (96%)	123 (96%)	54 (96%)	
>233.700	7 (3.8%)	5 (3.9%)	2 (3.6%)	
**Postoperative serum CA125**				0.322
≤13.800	139 (76%)	99 (77%)	40 (71%)	
>13.800	45 (24%)	29 (23%)	16 (29%)	

^1^Median (IQR) or Frequency (%); ^2^ Wilcoxon rank sum test; Pearson’s Chi-squared test; Fisher’s exact test; ^3^EEC:endometrioid endometrial carcinoma; ^4^NEEC:non-endometrioid endometrial carcinoma; ^5^NLR: neutrophil-lymphocyte ratio.

Bold values in the table represent the clinical factors included in the study.

### 3.2 Clinical characteristics screening and model construction

ROC curves were plotted to determine the optimal critical values for NLR, FIB, serum CA125, and postoperative serum CA125. The results of the univariate analysis indicated that several factors significantly increased the risk of recurrence in patients with EC. These factors included age, diabetes mellitus, FIGO stage, pathological grade, lymph node metastasis, radiotherapy, chemotherapy, FIB, serum CA125, and postoperative serum CA125, all of which had p-values less than 0.05. The factors identified were incorporated into a multivariate analysis to determine three clinically independent risk factors for pathological grade: p=0.009, FIB: p=0.036, and postoperative serum CA125: p=0.002 (**Table 2**). In parallel, the clinical model (CM) was developed. The results indicated that the mean AUC for the CM in the training set was 0.771 (95% CI: 0.693 - 0.894), while the AUC for the test set was 0.777 (95% CI: 0.622 - 0.933) (**Supplementary Figure S2**).

**Table 2:** Univariate and multivariate analysis results table.

Variable	Descript	P (univariable)	95% CI (univariable)	P (multivariable)	95% CI (multivariable)
**Age**	Mean ± SD	0.043	1.05 (1.00-1.10)	0.081	1.06 (0.99-1.13)
**Age at menarche**	Mean ± SD	0.995	1.00 (0.82-1.22)		
**Menopausal status**	No				
	Yes	0.152	1.76 (0.81-3.82)		
**Reproductive history**					
	Yes	0.338	0.26 (0.02-4.18)		
**Hypertensive**	No				
	Yes	0.718	1.14 (0.55-2.38)		
**Diabetes**	No				
	Yes	0.009	3.37 (1.36-8.35)	0.252	2.09 (0.59-7.39)
**F1GO stage**	I				
	II	0.861	1.09 (0.41-2.93)	0.613	0.70 (0.18-2.77)
	III	<0.001	39.33 (4.70-329.21)	0.303	4.28 (0.27-68.13)
**Pathological type**	NEEC^1^				
	EEC^2^	0.051	4.18 (0.99-17.55)		
**Pathological grade**	Low				
	High	<0.001	6.51 (2.69-15.78)	0.009	5.00 (1.49-16.72)
**Myometrial invasion**	None				
	<1/2	0.99	7335312.52 (0.00-NA)		
	≥1/2	0.99	36466982.23 (0.00-NA)		
**Lymph node metastasis**	No				
	Yes	<0.001	8.97 (3.37-23.86)	0.151	3.28 (0.65-16.63)
**Radiation therapy**	No				
	Yes	0.001	3.90 (1.72-8.82)	0.261	2.04 (0.59-7.03)
**Chemotherapy**	No				
	Yes	0.007	4.43 (1.49-13.18)	0.303	1.89 (0.56-6.33)
**Surgical procedure**	Laparoscopic surgery				
	Laparotomy	0.239	1.54 (0.75-3.16)		
**NLR** ^3^	≤1.765				
	>1.765	0.195	1.61 (0.78-3.30)		
**Fibrinogen**	≤3.790				
	>3.790	0.001	3.90 (1.72-8.82)	0.036	3.46 (1.08-11.08)
**Preoperative serum CA125**	≤233.700				
	>233.700	0.003	27.19 (3.16-233.71)	0.076	10.82 (0.78-150.90)
**Postoperative serum CA125**	≤13.800				
	>13.800	<0.001	5.38 (2.49-11.60)	0.002	4.97 (1.76-14.03)

^1^EEC, endometrioid endometrial carcinoma; ^2^NEEC, non-endometrioid endometrial carcinoma; ^3^NLR, neutrophil-lymphocyte ratio.

Bold values in the table represent the clinical factors included in the study.

### 3.3 Optimal classifier selection

**Figure 3:**
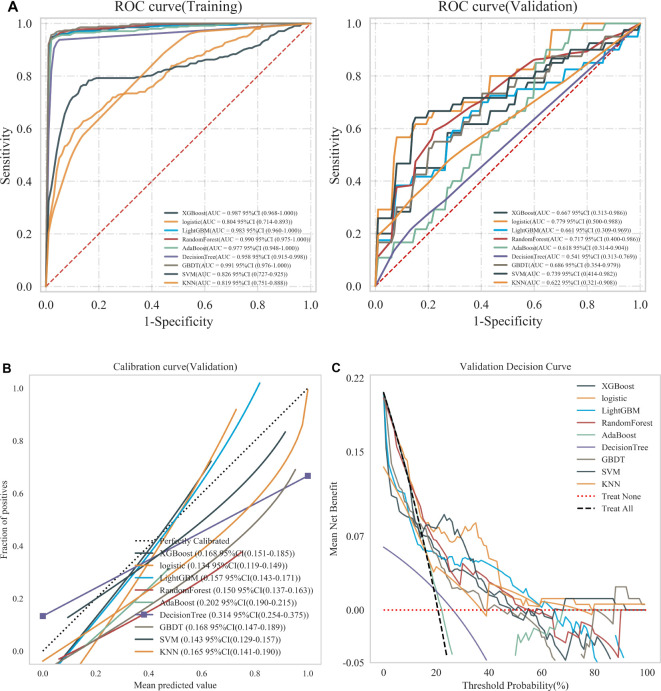
**(A)** ROC curves of nine machine learning classifiers in training and validation sets, **(B)** Calibration curves of nine machine learning classifiers in validation sets, and **(C)** DCA of nine machine learning classifiers in validation sets. Treat None: no action for all patients. Treat All: all patients were treated.

For each patient, features were extracted from the intratumoral, intratumoral with peritumoral 2mm, and intratumoral with peritumoral 4mm regions, resulting in 3,562, 7,124, and 7,124 features, respectively. These features underwent a process of downscaling and filtration (**Supplementary Figure S3**). This filtration identified 17 optimal intratumoral features, 15 optimal features for the intratumoral and peritumoral 2mm, and 12 for the intratumoral and peritumoral 4mm. The optimal intratumoral radiomics features were integrated into nine machine learning models to identify the most effective classifier for predicting recurrence in EC patients. **Figure 3** illustrates that the AUC for the nine classifiers in the training set ranges from 0.804 to 0.991, while in the validation set, the AUC ranges from 0.541 to 0.779. These results show that the AUC for LR has minimal variation between the training and validation sets, achieving the highest classification efficacy in the validation set with an AUC of 0.779 (95% CI: 0.500 to 0.988). Furthermore, the calibration curve for the validation set indicates that LR offers the best calibration performance at 0.134 (95% CI: 0.119 to 0.149). Additionally, in the decision curve analysis (DCA) of the validation set, LR demonstrates good clinical utility. Therefore, LR is selected as the optimal classifier for constructing the three radiomics models.

### 3.4 Radiomics model construction

**Figure 4:**
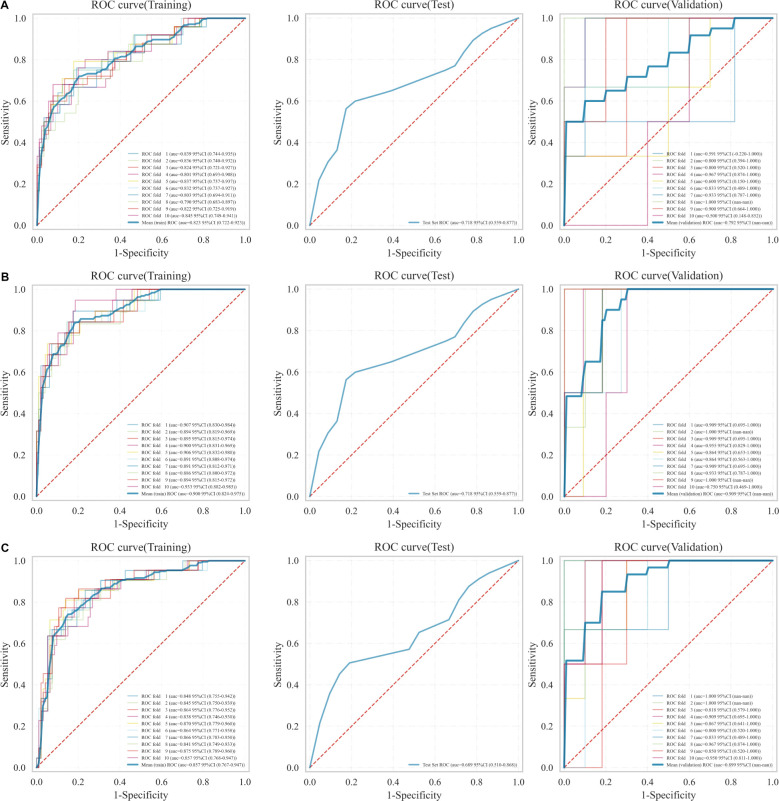
ROC curves for the three models in the training, test, and validation sets. **(A)** RM1, **(B)** RM2, **(C)** RM3.

The optimal radiomics features were incorporated into the LR classifier, and the model’s stability was verified through ten-fold nested cross-validation. The ROC curves were plotted to compare the models’ effectiveness in predicting recurrence in EC patients. The results, as illustrated in **Figure 4**, demonstrate that the RM2 achieved higher AUC values compared to the RM1 and the RM3 across the training, test, and validation sets. The RM2 demonstrated superior effectiveness in predicting the risk of recurrence in EC patients compared to the other two groups. Consequently, the Rad-score values of this model, in conjunction with the clinically independent risk factors, were selected for multivariate analysis (**Supplementary Table S1**).

### 3.5 Fusion model construction and evaluation

**Figure 5:**
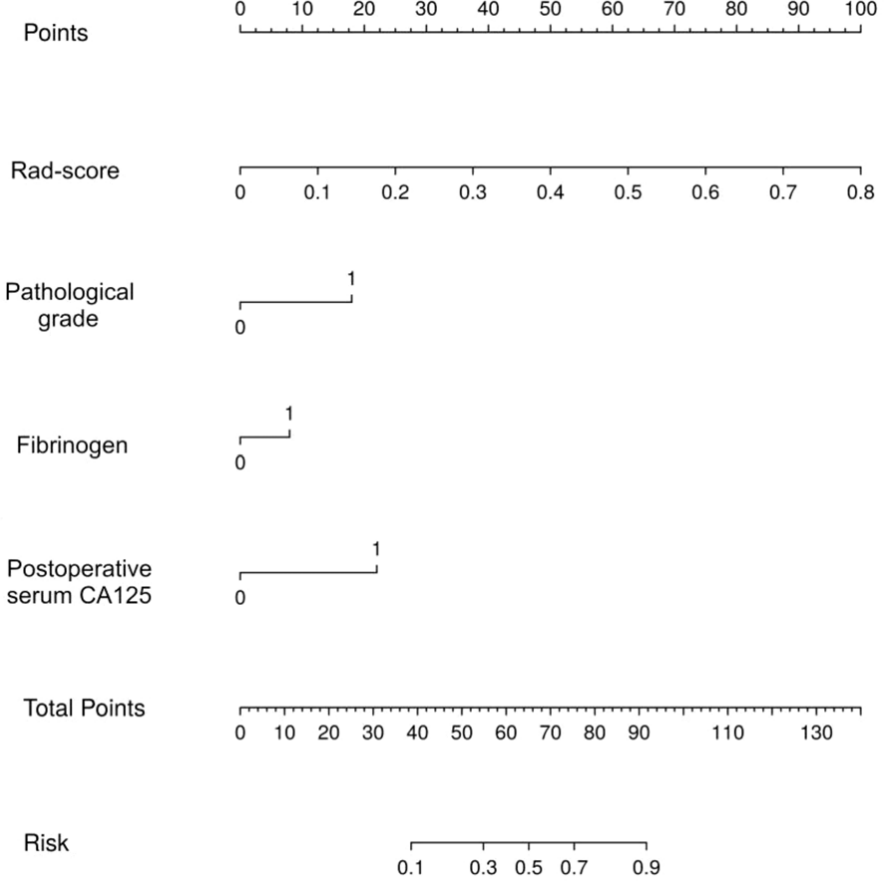
The nomogram for predicting the risk of recurrence in EC patients.

**Figure 6:**
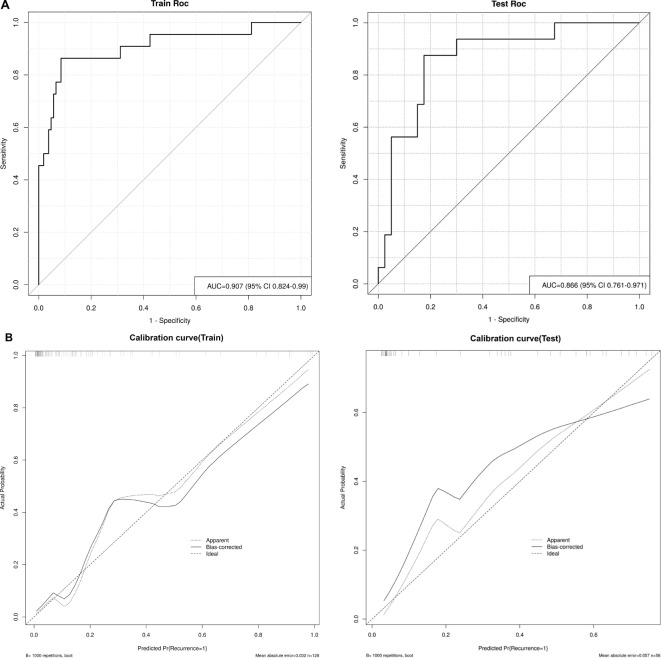
**(A)** ROC curves of the FM in the training and test sets, and **(B)** Calibration curves of the FM in the training and test sets. Apparent: empirical calibration curves. Bias-corrected: bias-corrected calibration curves. Ideal: perfect calibration curve.

**Figure 7:**
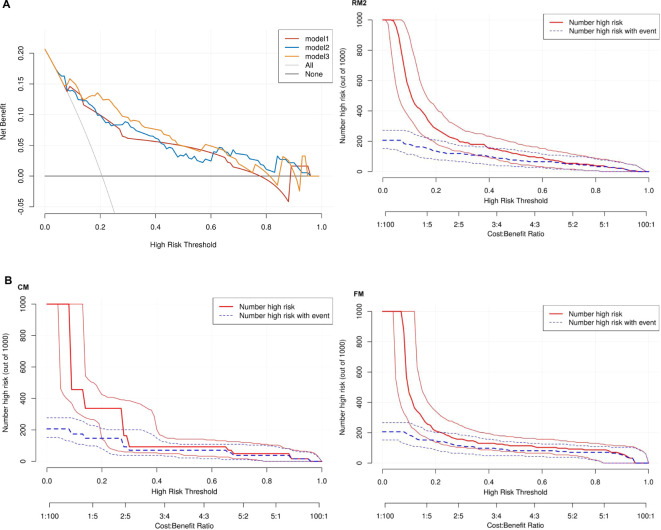
**(A)** Three models of DCA. Model 1: CM. Model 2: RM2. Model 3: FM. None: no action for all patients. All: all patients were treated. **(B)** CIC for CM, RM2 and FM.

Thereafter, the FM was constructed and plotted in a nomogram (**Figure 5**). As shown in **Figure 6**, the AUC of the FM is 0.907 (95% CI: 0.824 - 0.990) for the training set and 0.866 (95% CI: 0.761 - 0.971) for the test set. The calibration curves indicate that the observed curves closely align with the ideal curves, with Brier scores of 0.073 for the training set and 0.183 for the validation set. **Figure 7** presents DCA for the three models, indicating that all models provided greater net benefits compared to the no-action (Treat None) and full-treatment (Treat All) scenarios within a threshold probability range of 0.1 to 0.8. The RM2 showed enhanced clinical benefits for threshold probabilities exceeding 0.6. However, the FM demonstrated the most substantial advantages for clinical decision-making within the 0.1 to 0.6 threshold range. This model achieved the highest net benefit and most uniform variation, suggesting improved generalization and consistent performance. The CIC of the three models showed that FM had the largest agreement range, meaning that its clinical predictions and actual occurrences were highly consistent after the threshold value exceeded 0.2, reflecting higher clinical prediction efficiency. The DeLong test (**Table 3**) revealed statistically significant differences in predicting the risk of recurrence among the three models for EC patients (*p* < 0.05), indicating that the FM has superior diagnostic performance.

**Table 3:** Delong test p-value table.

Model	CM	RM2	FM
**CM**	—	0.72	0.036
**RM2**	0.72	—	0.014
**FM**	0.036	0.014	—

Bold values in the table represent the clinical factors included in the study.

### 3.6 Feature importance analysis

**Figure 8:**
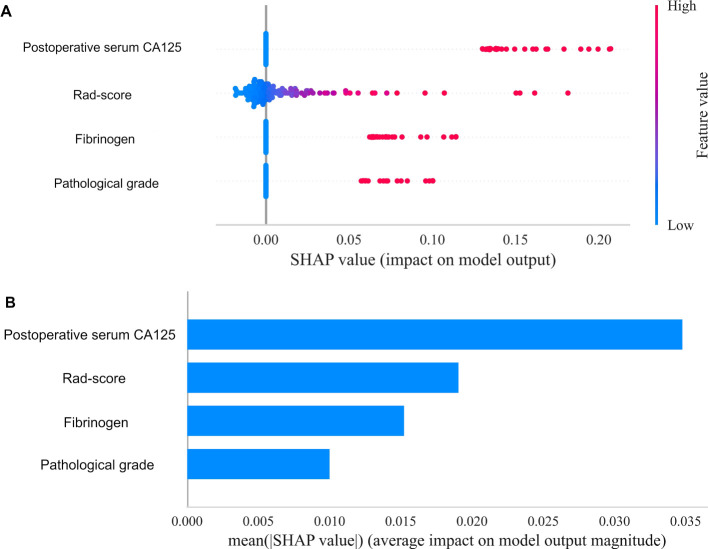
Overall visualization of the model through SHAP. **(A)** The SHAP beeswarm plot shows the positive or negative effects of each feature on the prediction probability through red and blue colors. **(B)** The SHAP bar chart shows the weight of the four most important characteristics in the model.

Using the SHapley Additive exPlanations (SHAP) methodology, we visualized the contributions of selected variables in predicting the risk of EC recurrence. **Figure 8A** displays the four most influential features in the model, while **Figure 8B** quantifies the feature-specific contributions using SHAP values along the X-axis. The results indicated that the Rad-score and postoperative serum CA125 had significantly higher contributions compared to other features, ranking as the top two predictors. This finding validates the effectiveness of integrating multi-sequence MRI peritumoral radiomics with clinical indicators. In contrast, FIB and pathological grade demonstrated lower contributions, suggesting that their predictive utility may be partially replaced by radiomics features in the integrated model. Elevated Rad-score values (indicated by the red distribution) were strongly associated with a higher risk of recurrence, highlighting the prognostic importance of both intratumoral and 2 mm peritumoral textural heterogeneity. An increase in postoperative CA125 consistently suggested a greater likelihood of recurrence, which aligns with its established role as a surveillance biomarker in the ESGO-ESTRO-ESP guidelines. In contrast, lower Rad-score values (represented by the blue distribution) were linked to low-risk profiles, which may indicate well-defined tumor boundaries and limited infiltration into the surrounding microenvironment.

## 4 Discussion

In this study, we found that LR outperforms the other eight machine learning classifiers in assessing recurrence in EC patients. The intratumoral and peritumoral 2mm radiomics model, constructed from 11 radiomics features, demonstrated superior diagnostic performance compared to the other two radiomics models. We developed and validated a CM, an RM based on multiple machine learning, and an FM constructed by combining clinical factors and a Rad-score to explore and validate the efficacy of the three models for predicting recurrence in EC patients. This model has the potential to significantly aid in clinical decision-making and personalized treatment for patients with EC.

In this study, the clinical characteristics were screened using univariate and multivariate analyses to identify three independent risk factors: pathological grade, FIB, and postoperative serum CA125. These findings are consistent with previous studies (13). Patients with a high pathological grade tend to be accompanied by a worse prognosis and are one of the most important indicators for determining the extent at the time of surgery (12), Lee et al. (14) showed that pathological grade, transvaginal ultrasound, and serum CA125 levels accurately predicted lymph node metastasis in patients with EC. There is a complex interplay between FIB and cancer growth and metastasis (15), with tumor cell invasion and development destroying procoagulant substances in human blood, and the body’s coagulation system is activated, resulting in elevated FIB levels. An increasing number of researchers have included laboratory markers in their analyses to explore their role in the prognosis of EC. Li (16) and others found that a nomogram based on preoperative FIB and other factors accurately predicted progression-free survival and overall survival in EC patients; one study (17) combined serum CA125 and FIB to construct a model for predicting the occurrence of vascular infiltration in EC patients and pointed out that FIB ≥2.78 was the best cutoff value for predicting vascular infiltration. Although serum CA125 is not a specific tumor marker and elevated levels may be associated with non-neoplastic diseases, many studies have demonstrated that changes in its level play an important role in prognostic assessment (18–20). Postoperative serum CA125 is defined as the level of serum CA125 in patients within 6–12 months after surgery, and few studies have included postoperative serum CA125 in the prognostic assessment of tumors. Wu et al. (21) found that this index was an independent risk factor for predicting recurrence in EC patients, with an optimal threshold of 13.75 U/mL. This is highly consistent with the present study, further underscoring the role of postoperative serum CA125 in forecasting recurrence and progression-free survival in EC patients. These studies have demonstrated the value of pathologic grading, FIB, and postoperative serum CA125 in related research areas, and in the present study, these metrics were included as clinically independent risk factors in the construction of predictive models to predict recurrence in EC patients.

In this study, we constructed an RM2 using logistic regression that incorporated a total of 15 features, with 10 features derived from DWI and 5 from T2WI. The majority of the selected features came from DWI, underscoring its critical role in assessing the likelihood of EC recurrence. DWI is capable of discerning microstructural alterations in tumor tissues, with the GLCM_Autocorrelation derived from the image being a salient feature in predicting the risk of EC recurrence. Autocorrelation is the self-correlation between pixel pairs of the same gray-level intensity within a Gray-Level Co-occurrence Matrix (GLCM). High autocorrelation values typically indicate a uniform distribution of gray-level intensities in the image, while low values suggest non-uniformity and heterogeneity in intensity distribution. In our study, we found that autocorrelation was significantly higher in recurrent patients compared to non-recurrent patients. These findings suggest that the GLCM_Autocorrelation feature is crucial for enhancing the accuracy of tumor detection and classification. This aligns with the conclusions of Dheepak et al. (22), who also emphasized the significance of this feature in cancer analysis. In tumor biology, E-cadherin is a critical cell adhesion molecule whose expression level is closely correlated with the adhesive capacity between tumor cells. High E-cadherin expression is typically associated with strong intercellular adhesion and lower invasive potential, while low expression is linked to the epithelial-mesenchymal transition (EMT), a key mechanism underlying tumor recurrence and metastasis (23–25). In EC, low GLCM_Autocorrelation values may indicate disrupted intercellular adhesion and initiation of EMT, conferring elevated recurrence risk. This association can be validated through histopathological studies and immunohistochemical analyses, such as assessing E-cadherin expression and EMT-related markers (e.g., vimentin). The GLSZM_SmallAreaEmphasis extracted from the T2WI image has been identified as a critical feature for predicting the risk of EC recurrence. This measure quantifies the distribution of small-sized areas within the image, with larger values indicating a finer texture characterized by numerous small regions. The diagnostic and differential diagnostic value of this feature has been extensively investigated. Li et al. (26) applied this feature to differentiate ovarian granulosa cell tumors (OGCTs) from ovarian fibroma-fibrosarcoma (OTCA-FTCA). Their findings indicated that OTCA-FTCA exhibited significantly lower values of this feature compared to OGCTs, suggesting the presence of smaller areas and finer textures in the solid lesions of OTCA-FTCA. Our study shows that this feature plays an important role in predicting recurrence in EC patients, highlighting its potential as a significant predictor in various cancer types.

The efficacy of machine learning algorithms largely depends on their application in optimal scenarios, and the performance of models constructed using these algorithms can differ significantly. To date, no comprehensive study has examined the most suitable machine learning algorithm for predicting the recurrence of EC or the disease-free survival of EC patients. While some research has explored the use of specific algorithms, a broader analysis comparing various algorithms is still necessary to identify the best approach for EC prediction (11, 27, 28). While most existing studies rely on a single classifier for model construction, this research incorporates intratumoral radiomics features into eight different machine learning classifiers to predict recurrence in EC patients. The findings indicate that LR is the most effective machine learning classifier, suitable for binary classification problems, and exhibits enhanced stability and robustness. Furthermore, it is easily extensible and modifiable. This study serves as a valuable reference for future research efforts.

Most existing MRI-based studies on EC radiomics have primarily focused on analyzing the intratumoral region (29–31). However, as research on malignant tumors advances, tumor invasion often infiltrates surrounding normal tissues, providing useful information in the peritumoral region that reflects tumor progression and prognostic information (32). Studies have shown that peritumoral radiomics can effectively predict EC lymph node metastasis and deep myometrial invasion (33, 34). The selection of 2mm and 4mm peritumoral regions was based on the unique pathophysiology of EC. The depth of myometrial invasion is a known prognostic factor, with microenvironmental changes, such as angiogenesis and immune infiltration, typically extending 3-5mm beyond the tumor boundary according to histopathology studies (35, 36). To capture both immediate peritumoral stroma (≤2mm) and intermediate-range microenvironment (≤4mm), we empirically tested these biologically plausible distances. Previous radiomics studies in solid tumors suggest that peritumoral regions within 5mm often contain prognostically relevant heterogeneity (11, 37, 38). For instance, Lin et al. (39) constructed various radiomics models that combined intratumoral and peritumoral data from 3mm, 5mm, and 10mm peritumoral regions derived from contrast-enhanced MR images to preoperatively predict treatment responses to transarterial chemoembolization in patients with hepatocellular carcinoma. Specifically, in the context of EC, Lin et al. (11) utilized 3mm and 5mm peritumoral expansions for predicting recurrence, but they did not compare multiple ranges. To address this gap, we systematically evaluated 2mm and 4mm as candidate thresholds.

In our study, we developed three radiomics models. Notably, the model that incorporated the intratumoral region along with a 2 mm peritumoral margin demonstrated the highest predictive efficacy. According to the literature (40, 41), the 2 mm peritumoral region may more accurately reflect the tumor invasion front and the biological changes occurring in the tumor microenvironment. In contrast, the 4 mm peritumoral areas likely contain more non-specific stromal components, which can dilute the biological signatures associated with recurrence. As a result, the RM2 model is better than the RM3 model at capturing the microenvironmental features that are prognostic of tumor recurrence. Additionally, spatial heterogeneity plays a critical role in the tumor microenvironment. The 2 mm peritumoral region may effectively capture the interactions between the tumor and stroma that drive invasion and recurrence. For example (42, 43), this zone is likely to have higher densities of cancer-associated fibroblasts (CAFs) and immune cell infiltration, both of which are essential in mediating tumor aggressiveness and the likelihood of recurrence. This finding contrasts with a similar study conducted by Lin et al. (11), where an intratumoral-based radiomics model exhibited optimal predictive performance. In their study, the peritumoral region did not provide additional information for EC recurrence. This discrepancy may be attributed to the fact that radiomics features derived from different MRI sequences contain different predictive information regarding the recurrence of EC. While it is true that excessive expansion of the peritumoral ROI can include too much normal tissue and obscure the potential recurrence information from the tumor region, expanding the peritumoral ROI to 2 mm, compared to 1mm, allows for the inclusion of both more normal tissue and more tumor-related information. This provides a valuable reference for future studies aiming to expand the boundaries of the peritumoral region. There is currently no consensus on the optimal size for this region. Ding et al. (44) systematically investigated how the size of the peritumoral region affects prediction performance in radiomics. Their findings indicate that the selection of peritumoral size depends on the ROI and significantly influences the final prediction performance of the radiomics model. These results suggest that peritumoral features should be optimized in future radiomics studies. Another study (45) explored the predictive performance of different radiomics models for predicting vascular invasion, myometrial invasion, and pathological staging in EC. The results showed that intratumoral and peritumoral features can provide complementary information for the comprehensive prognosis of EC, which is consistent with the results of the current study.

The present study is not without its limitations. Firstly, it is a single-center study with a limited sample size, which may introduce bias to the results. To address this limitation, future research should include multi-center and larger-scale studies. Secondly, long-term single-site data may still carry unmeasurable temporal variations. In future work, while integrating data from different periods, we will attempt to minimize the impact of temporal bias. Thirdly, the study utilized DWI sequences and T2WI sequences from MRI, which are limited in terms of image sequences. Incorporating additional sequences in future studies is expected to enhance the predictive capacity of the radiomics model. Fourthly, despite the delineation of the 3D ROI in this study, some discontinuities were observed at the image level in a limited number of patients during the transfer of images to the Darwin Research Platform for ROI segmentation, which may have an impact on the model’s prediction performance. Fifthly, the peritumoral ROI was obtained by automatically expanding the focal area by 2 mm and 4 mm, respectively. However, the optimal peritumoral range remains to be elucidated, and the range refinement score can be explored in the future. Lastly, this study focused solely on patient recurrence, without considering the recurrence site. A more detailed stratification of recurrence locations could lead to more targeted predictions and treatment recommendations.

In conclusion, the nomogram model, constructed based on intratumoral and peritumoral 2mm radiomics combined with clinical factors, can effectively predict the risk of recurrence in EC patients. This model provides a scientific basis for clinical decision-making and personalized patient treatment.

## Data Availability

The original contributions presented in the study are included in the article/**Supplementary Material**. Further inquiries can be directed to the corresponding author.
