# Supplementary material for: Predicting recurrence risk in endometrial cancer: a multisequence MRI intratumoral and peritumoral radiomics nomogram approach
Source: Front Oncol. 2025 May 6;15:1569729. doi: 10.3389/fonc.2025.1569729 (PMC12088970; doi:10.3389/fonc.2025.1569729)
Supplement: Supplementary file 1 [file DataSheet1.docx]

**Electronic Supplementary Material**

**Appendix E1**

**Follow-up and Evaluation Indicators**

Patients were followed up for a minimum of 36 months, primarily through clinical visits and telephone consultations. Follow-up visits were typically conducted every three to six months during the first two years after surgery, every six months for the subsequent three to five years, and annually from the sixth year onwards. The follow-up included a comprehensive medical history, physical examination, routine blood tests, comprehensive biochemistry and tumor marker tests, and imaging tests if deemed necessary. In this context, recurrence is defined as the reappearance of a tumor lesion at any site in patients with EC who have been disease-free for more than six months following initial treatment.

**Appendix E2**

**Image Acquisition**

The MRI was performed using a 3.0 T MRI scanner (Philips Achieva, The Netherlands). The MRI scanning sequences and parameters are as follows: (1) T2WI sequence, with a repetition time (TR) of 5000 ms, echo time (TE) of 60 ms, slice thickness of 5 mm, slice spacing of 1 mm, field of view (FOV) of 350 mm × 350 mm, and matrix size of 250 × 280; (2) DWI sequence, with a TR of 2750 ms, TE of 50 ms, slice thickness of 5 mm, slice spacing of 1 mm, FOV of 375 mm × 300 mm, matrix size of 125 × 150, and b-values of 0 and 800 s/mm². All images were saved in DICOM format.

**Table S1**: Training set multivariate logistic regression analysis.

| **Variable** | **Descript** | **P (multivariable)** | **95% CI (multivariable)** |
| --- | --- | --- | --- |
| **Pathological grade** | Low |  |  |
|  | High | 0.013 | 4.29（1.35 - 13.61） |
| **Fibrinogen** | ≤3.790 |  |  |
|  | ＞3.790 | 0.005 | 4.72（1.58 - 14.13） |
| **Postoperative serum CA125** | ≤13.800 |  |  |
|  | ＞13.800 | <0.001 | 6.28（2.23 - 17.64） |
| **Rad-score** | Mean ± SD | <0.001 | 1607.51（55.08 - 46917.32） |


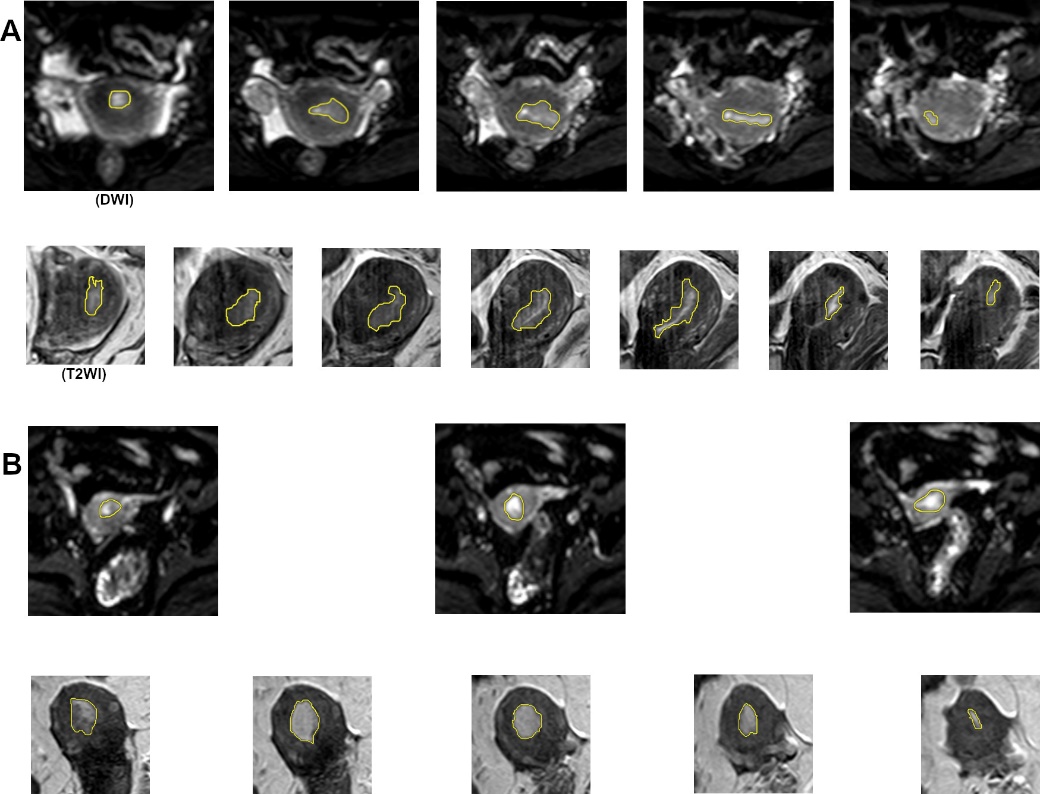


Figure S1: Tumor segmentation on T2WI and DWI images on the Darwin platform. The upper panel(A)presents a 46-year-old patient diagnosed with non-recurrent EC, whereas the lower panel(B)shows a 72-year-old patient with recurrent EC. In both images, the ROI is carefully delineated in layers. The platform then automatically expands this region by 2 mm and 4 mm to define the peritumoral ROI. Subsequently, following the completion of the outlining process, the 3D radiomics features are meticulously extracted for further analysis.


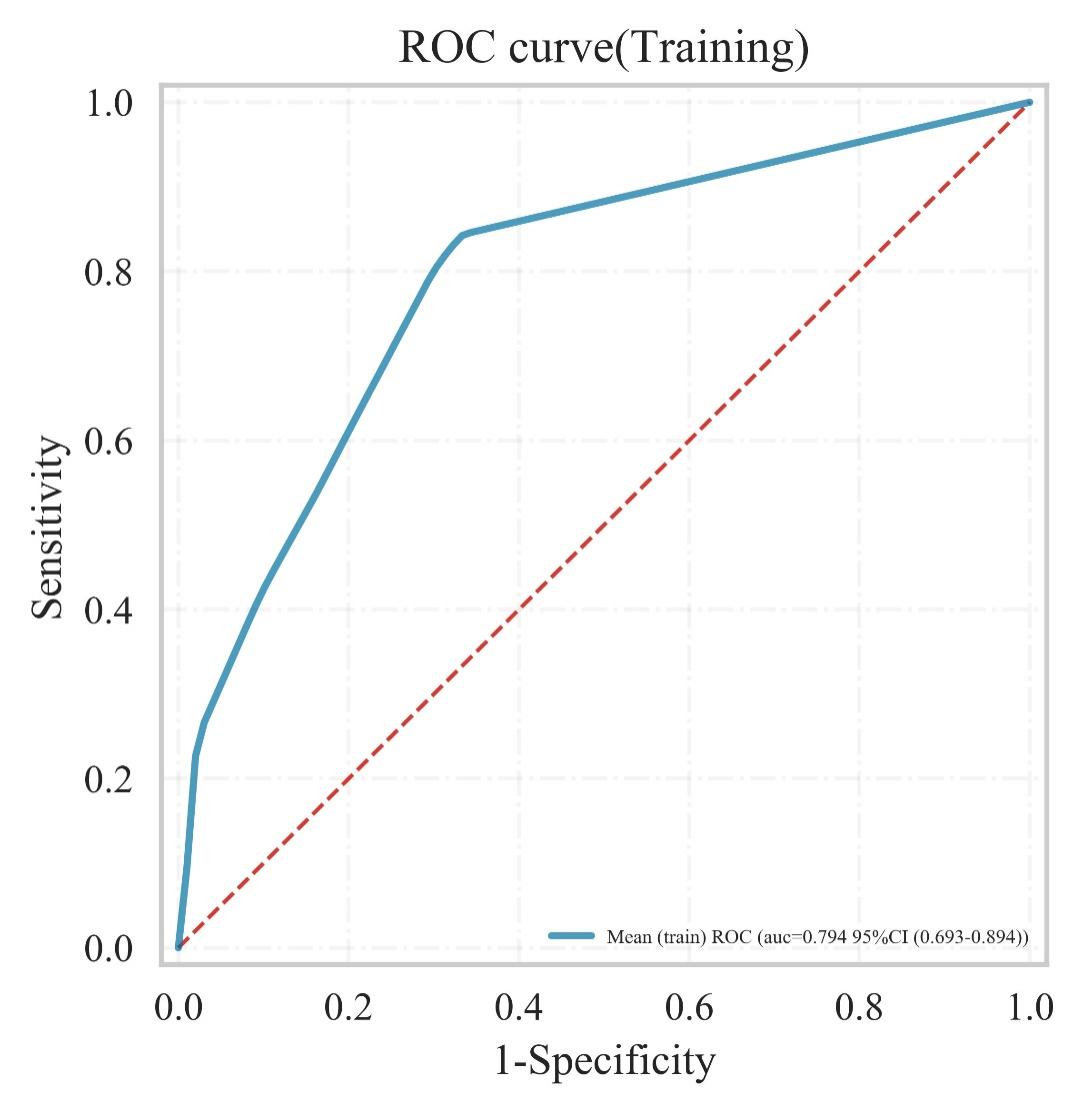


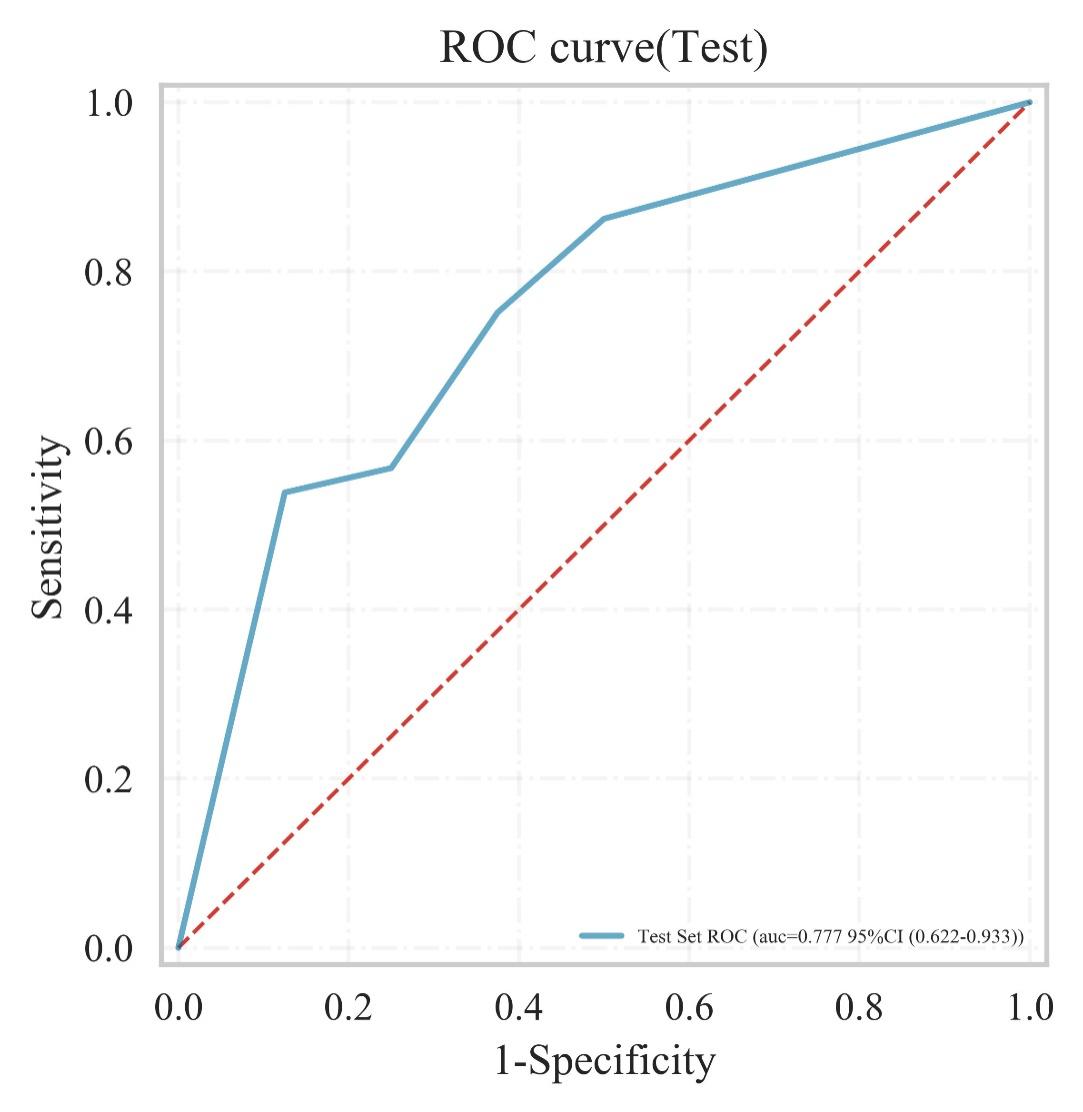


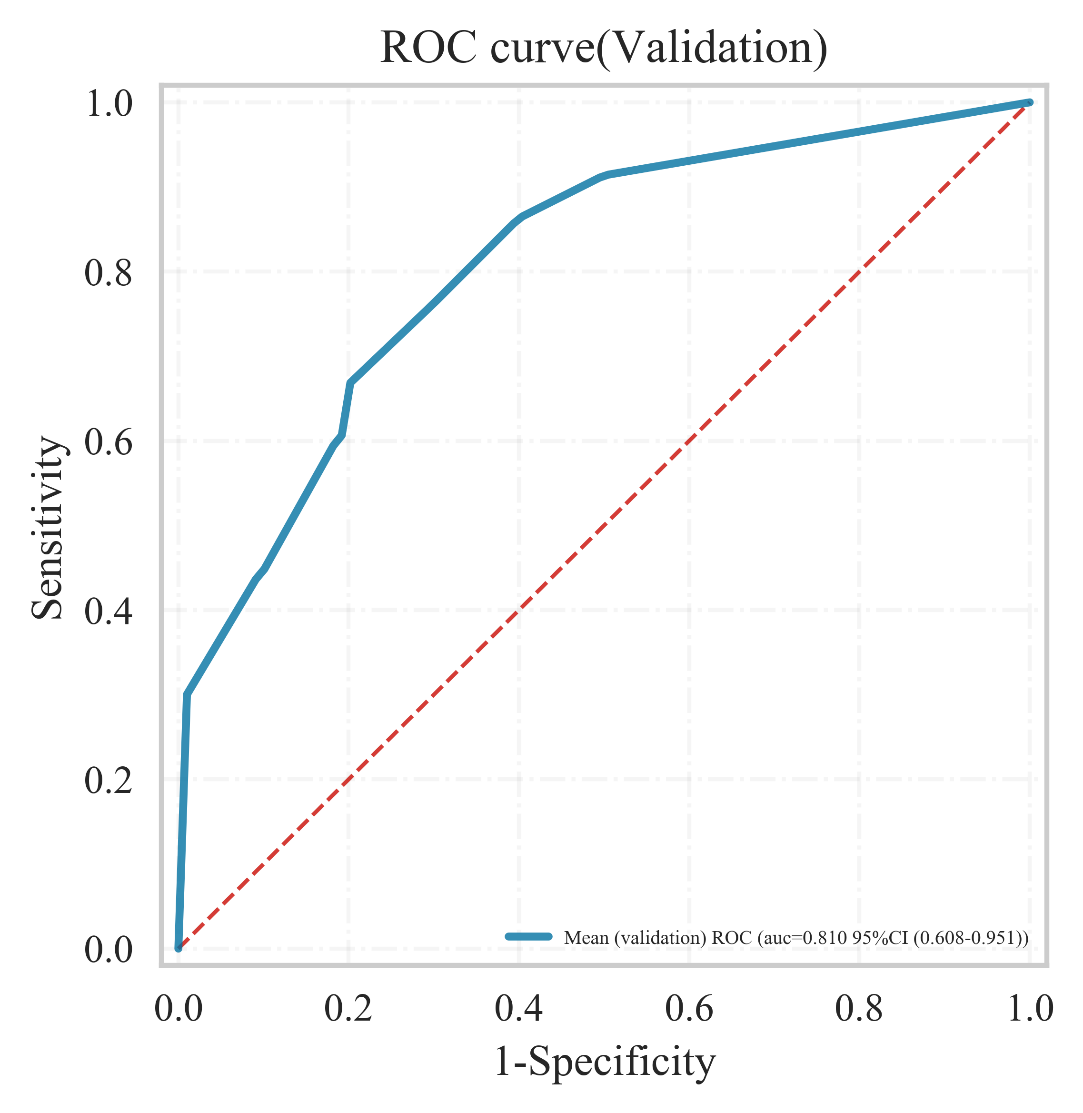


Figure S2: The ROC curves of the CM in the training, test and validation sets.

(A)


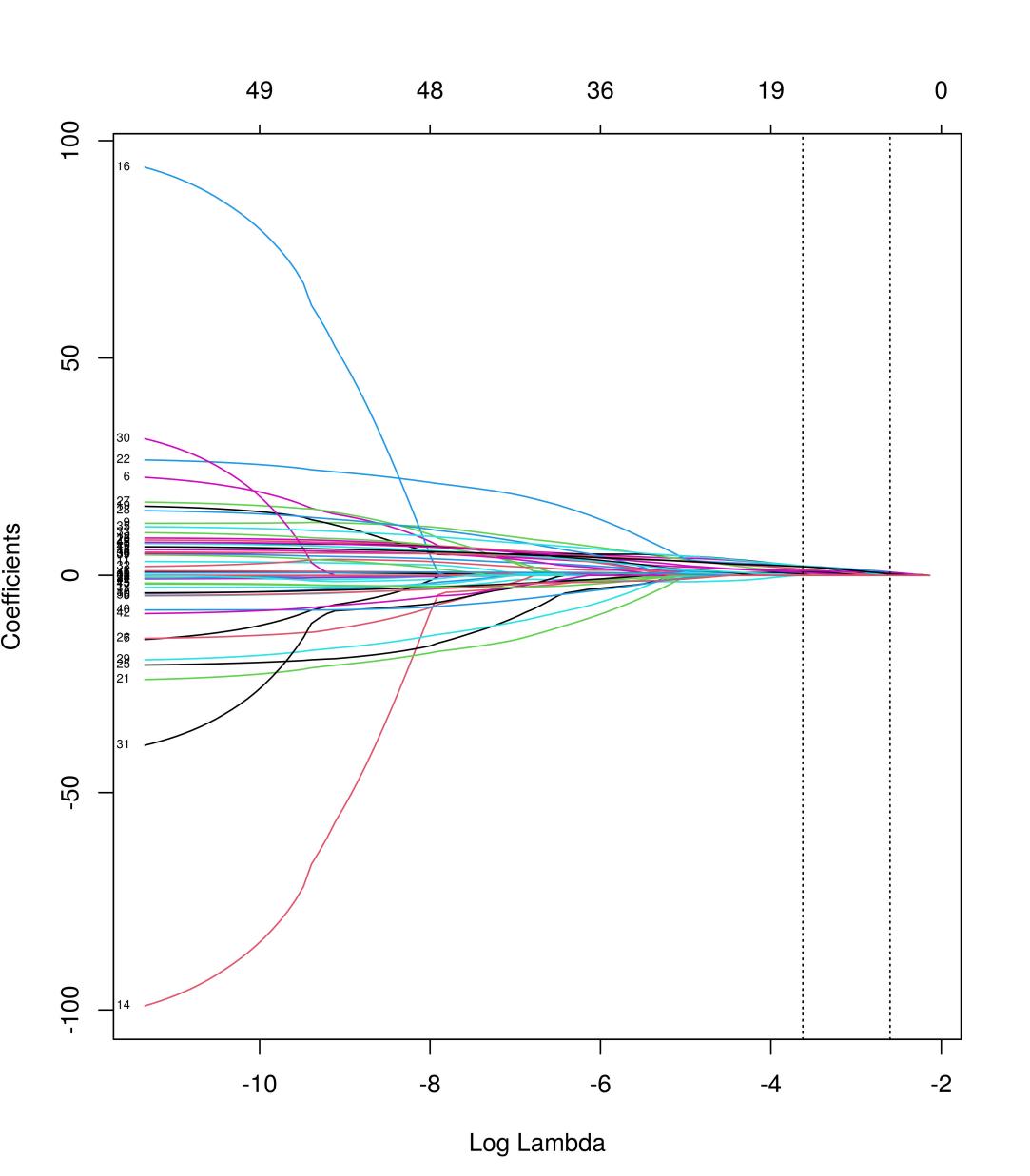


(B)


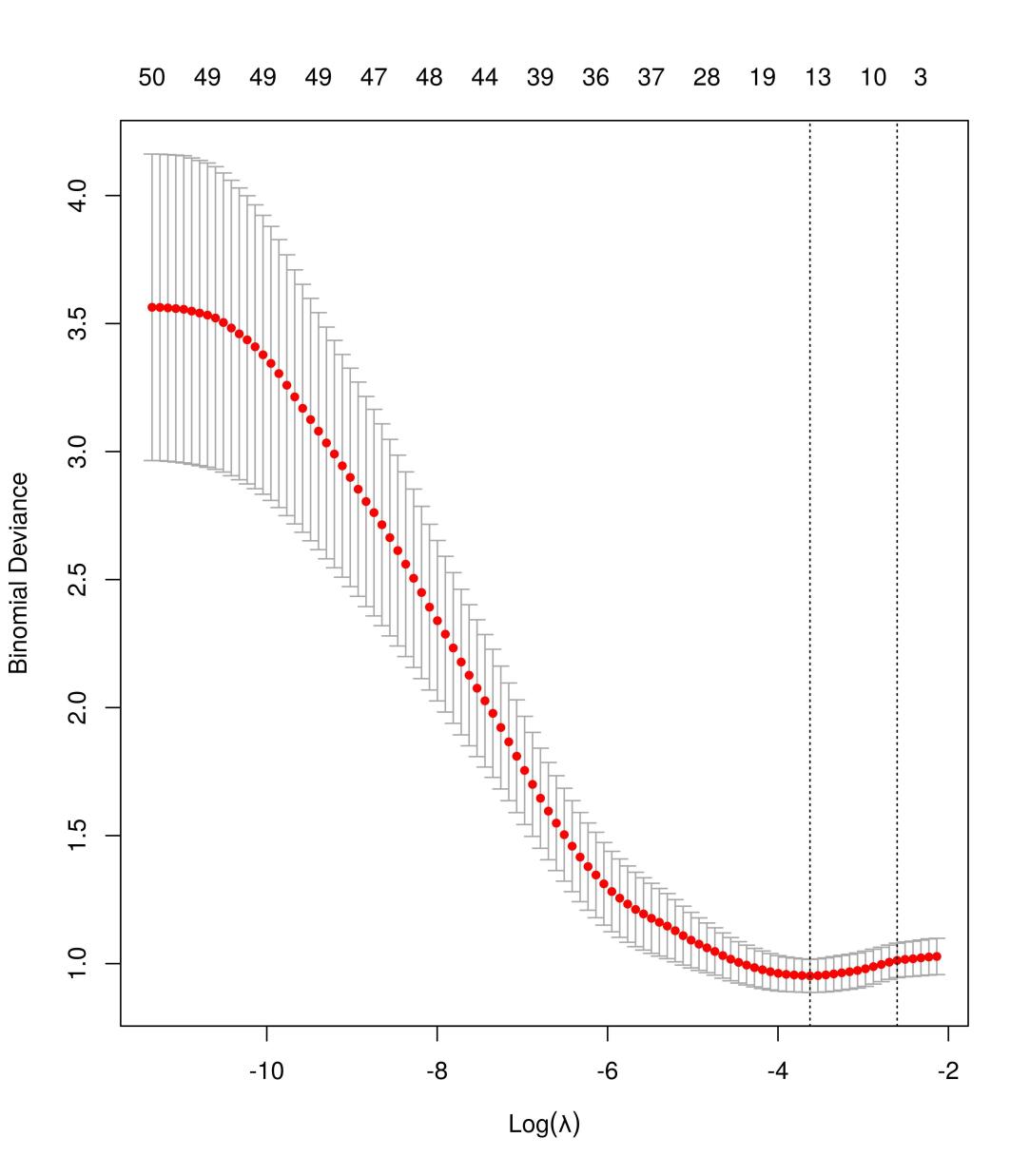


Figure S3: (A) Lasso regression coefficient paths for RM2 radiomics features. (B) Binomial deviance plot for RM2 radiomics features.
